# Structural insights into the reaction mechanism of *S*-adenosyl-L-homocysteine hydrolase

**DOI:** 10.1038/srep16641

**Published:** 2015-11-17

**Authors:** Yoshio Kusakabe, Masaaki Ishihara, Tomonobu Umeda, Daisuke Kuroda, Masayuki Nakanishi, Yukio Kitade, Hiroaki Gouda, Kazuo T. Nakamura, Nobutada Tanaka

**Affiliations:** 1 School of Pharmacy, Showa University, 1-5-8 Hatanodai, Shinagawa-ku, Tokyo 142-8555, Japan; 2 College of Pharmaceutical Sciences, Matsuyama University, 4-2 Bunkyo-cho, Matsuyama, Ehime 790-8578, Japan; 3 Faculty of Engineering, Gifu University, 1-1 Yanagido, Gifu, Gifu 501-1193, Japan

## Abstract

*S*-adenosyl-L-homocysteine hydrolase (SAH hydrolase or SAHH) is a highly conserved enzyme that catalyses the reversible hydrolysis of SAH to L-homocysteine (HCY) and adenosine (ADO). High-resolution crystal structures have been reported for bacterial and plant SAHHs, but not mammalian SAHHs. Here, we report the first high-resolution crystal structure of mammalian SAHH (mouse SAHH) in complex with a reaction product (ADO) and with two reaction intermediate analogues—3’-keto-aristeromycin (3KA) and noraristeromycin (NRN)—at resolutions of 1.55, 1.55, and 1.65 Å. Each of the three structures constitutes a structural snapshot of one of the last three steps of the five-step process of SAH hydrolysis by SAHH. In the NRN complex, a water molecule, which is an essential substrate for ADO formation, is structurally identified for the first time as the candidate donor in a Michael addition by SAHH to the 3’-keto-4’,5’-didehydroadenosine reaction intermediate. The presence of the water molecule is consistent with the reaction mechanism proposed by Palmer & Abeles in 1979. These results provide insights into the reaction mechanism of the SAHH enzyme.

*S*-adenosyl-L-methionine (SAM) is a widely used methyl donor^1^. Numerous methyl transferases transfer the methyl group from SAM to their respective cellular substrates^2^, including DNA, mRNA, rRNA, tRNA, histones H3 and H4, ribosomal proteins, and other proteins. The transfer produces *S*-adenosyl-L-homocysteine (SAH). SAH is hydrolysed by *S*-adenosyl-L-homocysteine hydrolase (SAHH, EC 3.3.1.1) to L-homocysteine (HCY) and adenosine (ADO). SAHH is one of the most highly conserved enzymes across kingdoms. SAHH dysfunction is associated with various diseases^2^, including vascular disorders^3^, myopathy^4^, fatty liver^5^, cancer^6^, renal insufficiency^7^ and diabetic nephropathy^8^. The molecular mass of each subunit of SAHH ranges from 45 to 55 kDa. The quaternary structures of SAHH can vary among species. The SAHH enzymes from human (HsSAHH)^9^, rat (RnSAHH)^10^, *Plasmodium falciparum* (PfSAHH)^11^, and *Mycobacterium tuberculosis* (MtSAHH)^12^ have been reported to form homotetramers, whereas the SAHH from the plant *Lupinus luteus* (LlSAHH)^13^ and the bacterium *Alcaligenesis faecalis*^14^ have been reported to form homodimers and homohexamers, respectively. Each SAHH monomer consists of two large domains (a cofactor-binding domain and a substrate-binding domain) that are separated by a cleft containing a deep pocket, two hinge regions, and a small C-terminal domain. Comparing the SAHH enzyme in the open form (RnSAHH)^10^ and closed form (HsSAHH)^9^ has revealed that the cleft between the two large domains closes upon substrate binding^10^. The protomer contains a tightly but not covalently bound endogenous NAD^+^ molecule in the crevice of the cofactor-binding domain. Recent high-resolution crystal structures of LlSAHH^13^ have revealed a sodium-binding site near the active site of each SAHH protomer, but the biological role of this bound cation is poorly understood. The inhibition of SAHH results in the cellular accumulation of SAH, which is a potent feedback inhibitor of SAM-dependent biological methylation. Because SAHH plays a key role in the regulation of transmethylation in all eukaryotic organisms, a number of SAHH inhibitors have been designed as drugs against various diseases, including cancer, malaria, tuberculosis, and viral infections^15^. Crystal structures of pathogenic SAHHs have been reported and used to design selective inhibitors against malaria (PfSAHH)^11^ and tuberculosis (MtSAHH)^12^. Over the past decade, inhibitors against mammalian SAHHs have also been shown to have immunosuppressive^16^ and plasma homocysteine-lowering effects^17^. Several crystal structures and biochemical studies have been reported for mammalian SAHHs^9^,^10^,^18^,^19^,^20^,^21^,^22^,^23^,^24^,^25^,^26^ and have helped to elucidate the reaction mechanism of SAHH. High-resolution structures of a bacterial SAHH (MtSAHH)^12^ and a plant SAHH (LlSAHH)^13^ have recently been reported, but the high-resolution structure of mammalian SAHH in complex with either inhibitor or substrate analogues has not (the resolution limits are currently 2.0 and 2.8 Å for HsSAHH and RnSAHH, respectively). In the absence of a high-resolution structure of mammalian SAHH complexed with an inhibitor (or substrate analogue), a complete structural understanding of the SAHH-substrate interactions has been impossible because understanding of the active-site water molecules is essential for fully understanding the reaction mechanism of mammalian SAHH. This knowledge will be important to guide the structure-based design of novel selective inhibitors of pathogenic or mammalian SAHHs. It is clear that high-resolution structural analysis of mammalian SAHH is of broad interest.

Here, we report the high-resolution crystal structures of mouse (*Mus musculus*) SAHH (MmSAHH) complexed with ADO, 3′-keto-aristeromycin (3KA), and noraristeromycin (NRN) at resolutions of 1.55, 1.55, and 1.65 Å, respectively. These results provide insights into the catalytic mechanism of SAHH. The structures also reveal a sodium cation near the active site. In addition, we have determined a 1.60-Å-resolution crystal structure of MmSAHH complexed with ribavirin (RBV), which is a potent inhibitor of SAHH^27^,^28^. Although RBV is a well-known guanosine analogue^29^, our crystal structure analysis clearly shows that RBV can also act as an adenosine analogue.

## Results

### Structure determination and overall structure of MmSAHH

**Figure 1:**
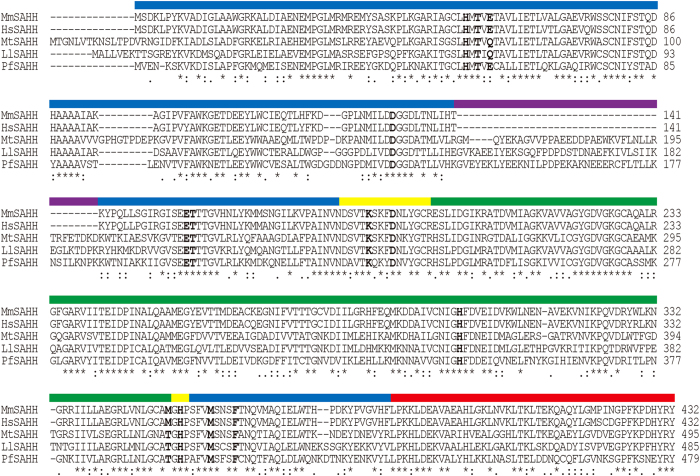
Amino acid sequence alignment of SAHHs. Residues involved in nucleoside binding in MmSAHH are highlighted. The coloured lines above the sequence alignment represent the domains in MmSAHH. The domains are coloured for the catalytic (blue), coenzyme-binding (green), hinge (yellow), and C-terminal (red) domains. Insertion segments of 40 amino acid residues exist in MtSAHH, LlSAHH, and PfSAHH but not in mammalian SAHHs are indicated by a purple line. The abbreviations used are as follows: MmSAHH, *Mus musculus* SAHH; HsSAHH, *Homo sapiens* SAHH; MtSAHH, *Mycobacterium tuberculosis* SAHH; LlSAHH, *Lupinus leteus* SAHH; and PfSAHH, *Plasmodium falciparum* SAHH.

**Figure 2:**
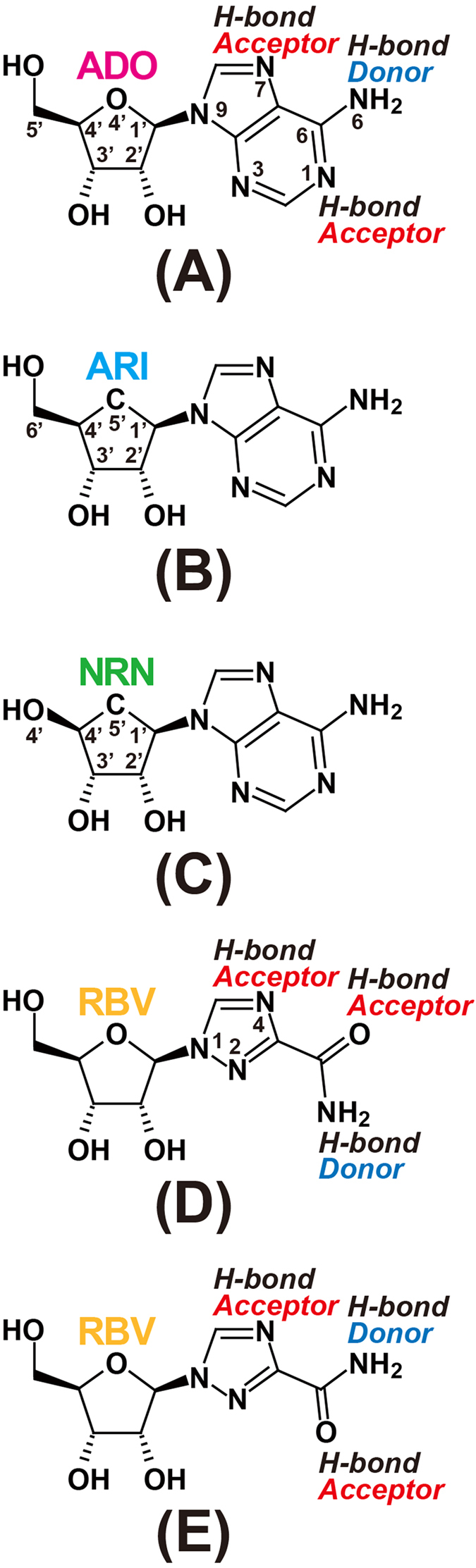
Nucleosides used in this study. (**A**) Adenosine (ADO). (**B**) Aristeromycin (ARI). (**C**) Noraristeromycin (NRN). (**D**) Ribavirin (RBV, drawn as a guanosine analogue). (**E**) Ribavirin (drawn as the adenosine analogue observed in this study).

**Figure 3:**
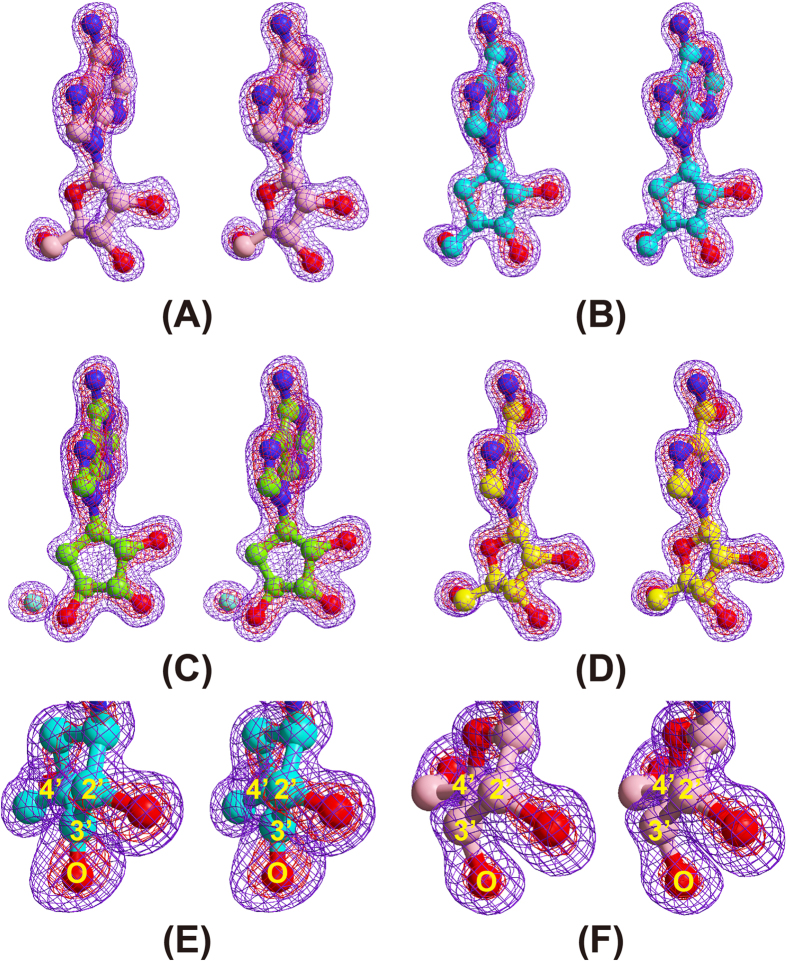
Wall-eyed pair stereo diagrams showing the |*Fo*| – |*Fc*| omit map of the bound nucleosides in the active site of MmSAHH. To exclude model bias, the structures were refined in the absence of the nucleoside molecule before map calculation. The amplitude |*F*c| and the phase angle calculated from the partial structure were then used to calculate the |*F*o| – |*F*c| omit map. The contour levels are 4.0 σ (violet) and 8.0 σ (red). (**A**) ADO at 1.55-Å resolution. (**B**) 3KA at 1.55-Å resolution. (**C**) NRN and a water molecule (see text) at 1.65-Å resolution. (**D**) RBV at 1.60-Å resolution. Comparison of (**E**) the electron density around the 3′-sp^2^-carbon of 3KA at 1.55-Å resolution and (**F**) that of the 3′-sp^3^-carbon of ADO at 1.55-Å resolution. Note that the 3′-C, 3′-O, 2′-C, and 4′-C atoms of 3KA are in the same plane.

**Figure 4:**
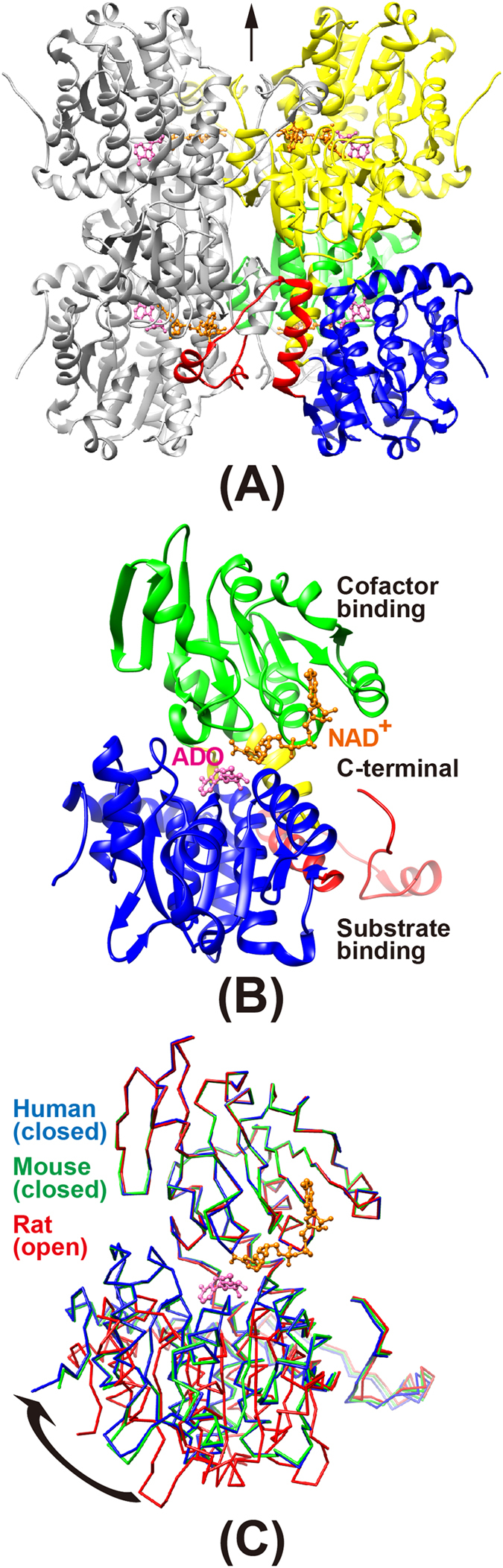
Overall structure of the MmSAHH/NAD^+^/ADO complex. (**A**) The MmSAHH tetramer, showing the catalytic (blue), coenzyme-binding (green), hinge (yellow), and C-terminal (red) domains of subunit A. Subunit B is shown in yellow. The molecules of bound NAD^+^ (orange) and ADO (pink) are shown as ball-and-stick models. Symmetry-related molecules in the *I*222 crystal are shown in grey. A crystallographic 2-fold axis is indicated by an arrow and coincides with one of the non-crystallographic 2-fold axis of the *222* symmetry of the tetramer. (**B**) The MmSAHH protomer coloured as in (**A**). The substrate-binding, cofactor-binding, and C-terminal domains are marked. (**C**) A comparison of the crystal structure of the ternary (Enzyme/NAD^+^/ADO closed form) complex of MmSAHH (green) with those of the ternary (Enzyme/NADH/3′-keto neplanocin A closed form) complex of HsSAHH (blue, PDB: 1LI4) and the binary (Enzyme/NAD^+^ open form) complex of RnSAHH (red, PDB: 1KY4). The bound ligands in HsSAHH and RnSAHH have been removed for clarity. Least-square fittings were done with respect to structurally equivalent 155 Ca atoms in the cofactor-binding domain of each molecule. The RMSD was 0.27 Å for MmSAHH vs. HsSAHH and 0.43 Å for MmSAHH vs. RnSAHH.

The MmSAHH enzyme forms a homotetramer, with each subunit consisting of 432 amino acid residues (Fig. 1) and a tightly but not covalently bound NAD^+^ cofactor. The molecular mass of each subunit is approximately 48 kDa. The crystal structures of MmSAHH complexed with adenosine (ADO), 3′-keto-aristeromycin (3KA), noraristeromycin (NRN), and ribavirin (RBV) (Fig. 2) were determined at resolutions of 1.55, 1.55, 1.65, and 1.60 Å, respectively (Fig. 3 and Tables S1 and S2). The SAHH crystal has a *222* point group symmetry with two subunits of MmSAHH in the asymmetric unit. They are related by a crystallographic 2-fold axis of symmetry to form the tetramer (Fig. 4A). The overall structure of MmSAHH is very similar to the structures of SAHHs from other species. For simplicity, the following description refers primarily to subunit A of the ADO complex. The secondary structure nomenclature is based on that of HsSAHH^9^. The subunit of MmSAHH consists of two large domains separated by a cleft containing a deep pocket, two hinge regions and a small C-terminal domain that is separate from the main body of the subunit and extends to the adjacent subunit (Fig. 4A,B). One of the large domains is responsible for cofactor binding, and the other is necessary for substrate binding. The two large domains are unequal in size; the substrate-binding domain is larger and comprises 212 residues, whereas the cofactor-binding domain comprises 155 residues.

### Substrate-binding domain

The substrate-binding domain comprises residues 1–181 and 355–385. It is an α/β-type structure consisting of eight α-helices and eight β-strands. The structural core in the domain is an eight-stranded parallel β-sheet in the centre of the domain that is sandwiched by two arrays of three α-helices each (Fig. 4B, blue). Insertion segments of approximately 40 amino acid residues were observed in the substrate-binding domains of PfSAHH^11^, MtSAHH^12^, and LlSAHH^13^, whereas these insertions do not exist in MmSAHH (Fig. 1), HsSAHH^9^ or RnSAHH^10^. The reaction product ADO (Fig. 4B, pink) was found in a crevice of the substrate-binding domain in each of the two subunits in the asymmetric unit of the MmSAHH crystal. The binding mode of the bound ligand molecules will be presented later.

### Cofactor-binding domain

The cofactor-binding domain comprises residues 197–351. The basic element of the secondary structure in this domain is a six-stranded parallel β-sheet in the centre of the domain that is sandwiched by two arrays of three α-helices each (Fig. 4B, green). The six-stranded parallel β-sheet is flanked by four α-helices and constitutes a characteristic dinucleotide-binding motif or Rossmann fold composed of two βαβαβ units. Although NAD^+^ was not exogenously added during the protein expression, purification, or crystallisation of MmSAHH, a tightly but not covalently bound endogenous NAD^+^ molecule (Fig. 4B, orange) was observed in a crevice of the cofactor-binding domain of each of the two subunits in the asymmetric unit of the MmSAHH crystal. The binding mode of the NAD^+^ molecule is very similar to those of SAHHs from other species.

### C-terminal domain

The C-terminal domain comprises residues 386–432 and has a helix-loop-helix structure (Fig. 4B, red). The domain extends to the adjacent subunit and covers the adenosine monophosphate moiety of the bound NAD^+^ molecule in the cofactor-binding domain of the adjacent subunit (Fig. 4A). The side chain of Lys426 forms bifurcated hydrogen bonds with O2′A and O3′A of the adenosine ribose of the NAD^+^ molecule. The Tyr430 side chain forms a hydrogen bond with the pyrophosphate oxygen on the adenine side of the NAD^+^ molecule. Similar inter-subunit interactions have also been reported for SAHHs from other species, and the Lys426 and Tyr430 residues are highly conserved in organisms including bacteria and mammals (Fig. 1).

### Conformational changes upon substrate binding

In the MmSAHH tetramer, the four cofactor-binding domains are located in the centre of the tetramer and form a rigid structural core. The substrate-binding domains are located far from the centre of the tetramer, and they have little interaction with each other. Two hinge regions—residues 182–196 (N-terminal hinge) and 352–354 (C-terminal hinge)—connect the two large domains (Fig. 4B, yellow). Accordingly, the substrate-binding domains are more mobile than the cofactor-binding domains. Superposition of the coordinate sets of the cofactor-binding domains of human and rat SAHHs onto the cofactor-binding domain of MmSAHH (Fig. 4C) shows that conformation of the substrate-binding domain of the mouse ternary complex (MmSAHH/NAD^+^/ADO) exhibits a closed conformation similar to that observed in the human ternary complex (HsSAHH/NADH/inhibitor)^21^ rather than the open conformation observed in the rat binary complex (RnSAHH/NAD^+^)^20^. The other (3KA, NRN, or RBV) complexes of MmSAHH also exhibit closed conformations. It is possible that the inter-domain cleft closes upon binding to ADO (or its analogues), although the crystal structure of the open form of MmSAHH has not yet been obtained. Recent crystal structures of SAHH from *Thermotoga maritima* (TmSAHH)^30^ have shown that the binary complex (TmSAHH/NAD^+^) adopts both open (space group *C*2 crystal) and closed (space group *P*3_1_21 crystal) conformations, despite the absence of bound nucleoside ligands. These observations indicate the flexible nature of the SAHH protomer and are consistent with the results from other crystal structure analyses of SAHHs from various species.

### ADO complex

**Figure 5:**
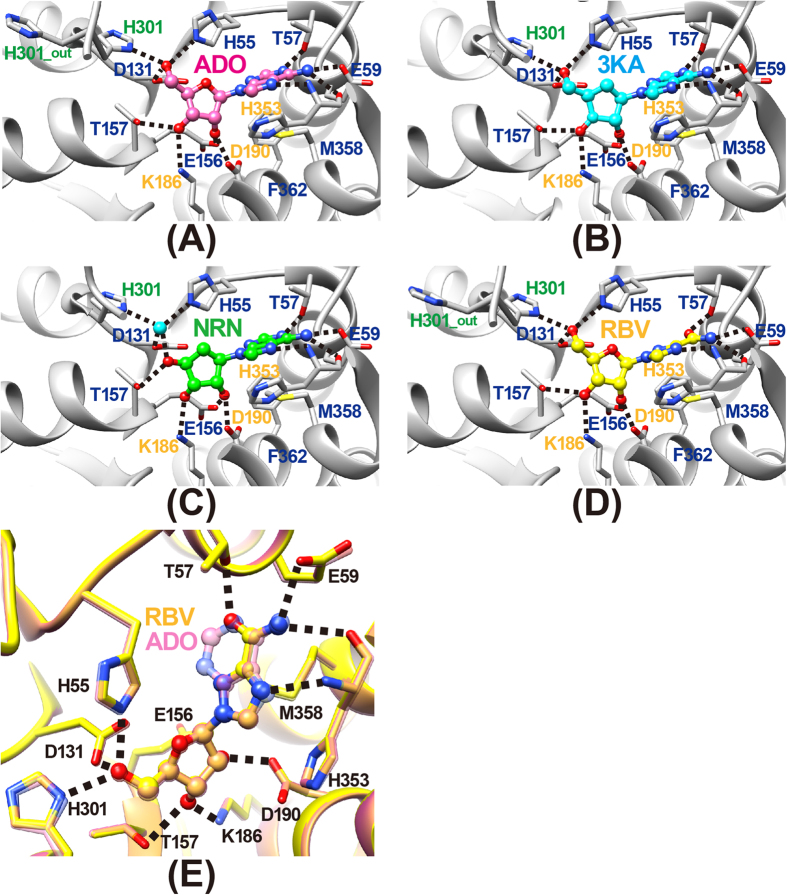
The mode of nucleoside binding in MmSAHH. Possible hydrogen bonds are indicated by dashed lines. (**A**) Adenosine (ADO). (**B**) 3′-Keto-aristeromycin (3KA). (**C**) Noraristeromycin (NRN). (**D**) Ribavirin (RBV). (**E**) A superposition of the ADO (transparent pink) and RBV (yellow) complexes. The flipped-out conformation of His301 observed in the ADO and RBV complexes is indicated as H301_out.

The binding mode of ADO (Fig. 2A) in MmSAHH is similar to that in SAHHs from other species. The ADO binds in a crevice of the substrate-binding domain (Fig. 5A). The ribose moiety of the ADO forms hydrogen bonds with the side chains of polar residues, including Glu156 and Asp190 (2′-OH), Thr157 and Lys186 (3′-OH), and His55, Asp131, and His301 (5′-OH). Notably, residues 300–304 exhibit dual conformations; some flip out (minor conformer, occupancy of 0.3), and others flip over (major conformer, occupancy of 0.7). In the flipped-out conformation, His301 flips out from the ADO-binding site and is displaced from the 5′-OH group of the bound ADO molecule. The adenine ring moiety of the ADO is accommodated by a hydrophobic pocket composed of Leu54 and Phe362 at the bottom and Leu347 and Met358 on the sides. The nitrogen atoms of the ADO (see Fig. 2A) are fixed by hydrogen bonds with the side chain of Thr57 (N1), the side chain of Glu59, the main-chain carbonyl group of His353 (N6) and the main-chain imino group of His353 (N7). ADO interacts with the residues belonging to the substrate-binding domain (Leu54, Thr57, Glu59, Asp131, Glu156, Thr157, Met358, and Phe362), as well as with those belonging to the hinge regions (Lys186, Asp190, and His353); the latter contribute to stabilising the closed conformation. Residues that are involved in hydrogen bonds with ADO are highly conserved among SAHHs from various species (Fig. 1). An exception is Glu59, which is replaced by Gln in the SAHHs of some species, including MtSAHH and LlSAHH.

### 3KA complex

ARI is a carbocyclic analogue of ADO; the ribosyl ring 4′-oxygen of ADO is replaced by a 5′-carbon atom in ARI (Fig. 2A,B). In this crystal structure analysis, co-crystallisation with ARI resulted in oxidation of the 3′-OH group of the nucleoside to form a 3′-keto-ARI (3KA). The omit electron density map of the nucleoside ligand clearly shows that the C3′ is planar, indicating that the O3′ has been oxidised to the keto form (Fig. 3E). This is the first direct evidence from a high-resolution (1.55 Å resolution) crystallographic structure showing the binding of a 3′-keto nucleoside to SAHH, although the binding of 3′-keto-ADO (2.8 Å resolution) and 3′-keto-ARI (2.0 Å resolution) have been reported for a mutant RnSAHH^18^ and MtSAHH^12^, respectively. The binding mode of 3KA in MmSAHH (Fig. 5B) is very similar to that of ADO described above, except that the flipped-out conformation of residues 300–304 in the ADO complex is not observed in the 3KA complex. Although the conformation of the 3′ position of the 3KA complex is different from that of the ADO complex (sp^2^ vs. sp^3^), the hydrogen bond network around the O3′ atom of the 3KA complex is very similar to that of the ADO complex. The hydrogen bond distances are 2.78 Å (Thr157-OG---O3′) and 2.83 Å (Lys186-NE---O3′) for the ADO complex, and 2.70 Å (Thr157-OG---O3′) and 2.82 Å (Lys186-NE---O3′) for the 3KA complex.

### NRN complex

NRN is a carbocyclic analogue of ADO that lacks a 5′-carbon (Fig. 2A,C). In other words, the 6′-CH_2_-OH group of ARI is replaced by the 4′–OH group in NRN (Fig. 2B,C). The MmSAHH complexed with NRN is the first crystal structure of SAHH to be solved with NRN. The binding mode of the adenine moiety of NRN in MmSAHH is very similar to that of ADO described above (Fig. 5C). However, there are notable differences in the recognition mode of the OH groups of NRN compared with ADO. The most remarkable observation is the presence of a water molecule adjacent to the 4′-OH group of NRN. The role of this water molecule in the reaction mechanism of SAHH will be fully described in the DISCUSSION section. The water molecule occupies a position equivalent to that occupied by the 5′-OH group in the ADO complex and forms hydrogen bonds with the side chains of His55, Asp131, and His301. As with 3KA complex, in the NRN complex, the flipped-out conformation of residues 300–304 is not observed. Another difference between the ADO and NRN complexes is the role of the Thr157 side chain. In the ADO and 3KA complexes, the OG atom of the side chain of Thr157 forms a hydrogen bond with the 3′-O atom of the nucleosides, whereas the side chain of Thr157 forms a hydrogen bond with the 4′-O atom of the nucleoside in the NRN complex. This difference is caused by a slight change in the torsion angle (χ) around the N-glycosidic bond from χ  =  249° for ADO to χ  =  232° for NRN. Nonetheless, the adenine rings of the nucleosides superpose very well. In the ADO complex, the OG atom of Thr157 is closer to the 3′-O atom (2.78 Å) than the 5′-C atom (3.52 Å). Conversely, in the NRN complex, the OG atom of Thr157 is closer to the 4′-O (structurally equivalent to 5′-C of ADO) atom (2.67 Å) than the 3′-O atom (3.39 Å).

### RBV complex

An antiviral drug RBV^29^ has been reported to be a potential inhibitor of HsSAHH^27^ and TcSAHH^28^. The MmSAHH complexed with RBV is the first crystal structure of SAHH to be solved with RBV (Fig. 5D). Interestingly, the bound RBV superposes very well onto the bound ADO (Fig. 5E). The hydrogen bond network around the base moiety of the RBV in MmSAHH is structurally equivalent to that of the ADO in MmSAHH. The N4 of the triazole ring of RBV spatially corresponds to the N7 of ADO (see Fig. 2A,E) and forms a hydrogen bond with the main-chain imino group of His353. The amide nitrogen of RBV spatially corresponds to the N6 of ADO (see Fig. 2A,E) and forms hydrogen bonds with the main-chain carbonyl group of His353 and the side chain of Glu59. The carbonyl oxygen of RBV spatially corresponds to the N1 of ADO and forms a hydrogen bond with the side chain of Thr57. Although RBV is a well-known guanosine analogue (Fig. 2D), this crystal structure clearly demonstrates that RBV can also act as an ADO analogue, because the hydrogen bond donors/acceptors are spatially conserved between ADO and RBV by rotation of the single bond between the triazole ring and carboxamide group of RBV (Fig. 2A,E). A hydrogen bond network around the ribose moiety of the RBV in MmSAHH is very similar to that of the ADO in MmSAHH. As in the ADO complex, residues 300–304 exhibit dual conformations in the RBV complex. The flipped-out (major) conformer has an occupancy of 0.6, and the flipped-over (minor) conformer has an occupancy of 0.4. In the RBV complex, the flipped-out conformation is the major conformer, whereas the flipped-over conformation is the major conformer in the ADO complex.

### Cation-binding site

**Figure 6:**
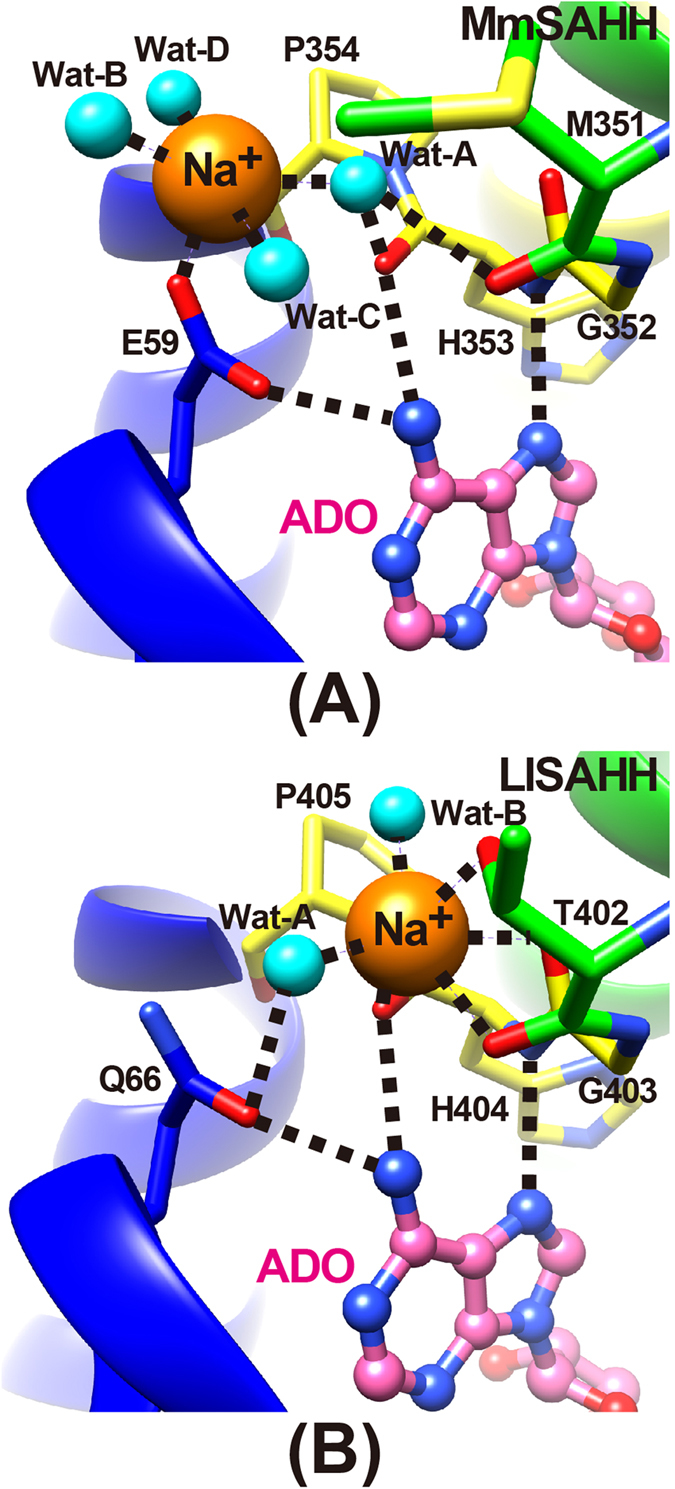
Cation-binding site of MmSAHH and LlSAHH. The bound Na^+^ cation is shown as an orange sphere, and water molecules are shown as cyan balls in the (**A**) MmSAHH/NAD^+^/ADO complex and (**B**) LlSAHH/NAD^+^/ADO complex. The protein residues are coloured as in Fig. 4A.

The high-resolution structures reported here enabled us to identify a sodium cation near the active site in the ADO, 3KA, and NRN complexes (Fig. 6A). One sodium cation was found at the C-terminal hinge region of each protomer. The cation-binding site in MmSAHH is similar to but distinct from those of plant LlSAHH^13^. In the active site of LlSAHH (Fig. 6B), the coordination geometry is a distorted octahedral geometry formed by three main-chain carbonyl groups (Thr402, Gly403, and His404), the side chain OH group of Thr402 and two water molecules. In the active site of MmSAHH, the bound sodium cation has a distorted trigonal-bipyramidal geometry and binds to the side chain of Glu59 and four water molecules. The side chain of Glu59 and two water molecules (Wat-A and Wat-B) are the equatorial ligands, and the axial ligands are the remaining two water molecules (Wat-C and Wat-D). The cation-ligand distances are 2.2 to 2.4 Å in all cases and are consistent with the typical Na-O (water or Asp/Glu) distances^31^,^32^ reported in the Cambridge Structural Database and Protein Data Bank. The Thr402 in LlSAHH is replaced by Met351 in MmSAHH, and the side chain of Met351 is not involved in the cation binding. This difference accounts for the change in the coordination geometry of cation binding between MmSAHH and LlSAHH. The Thr402 in LlSAHH is conserved in other SAHHs containing the 40 amino acid insert (e.g., PfSAHH, MtSAHH, and LlSAHH), whereas the Met351 in MmSAHH is conserved among mammalian SAHHs (Fig. 1). In any case, the bound cation appears to be involved in stabilising the C-terminal hinge region of the SAHH molecule, either directly (as in LlSAHH) or via a water molecule (as in MmSAHH). The cation contributes to the recognition of the adenine ring through hydrogen bonds formed by the main-chain NH- and C = O atoms of His353 (His404 in LlSAHH) in the C-terminal hinge. In the RBV complex of MmSAHH, the sodium cation was not observed in the active site. The change is caused by a different hydrogen bonding network in the RBV complex, in which the side chain of Glu59 exhibits a slightly different conformation than that in the ADO complex.

## Discussion

**Figure 7:**
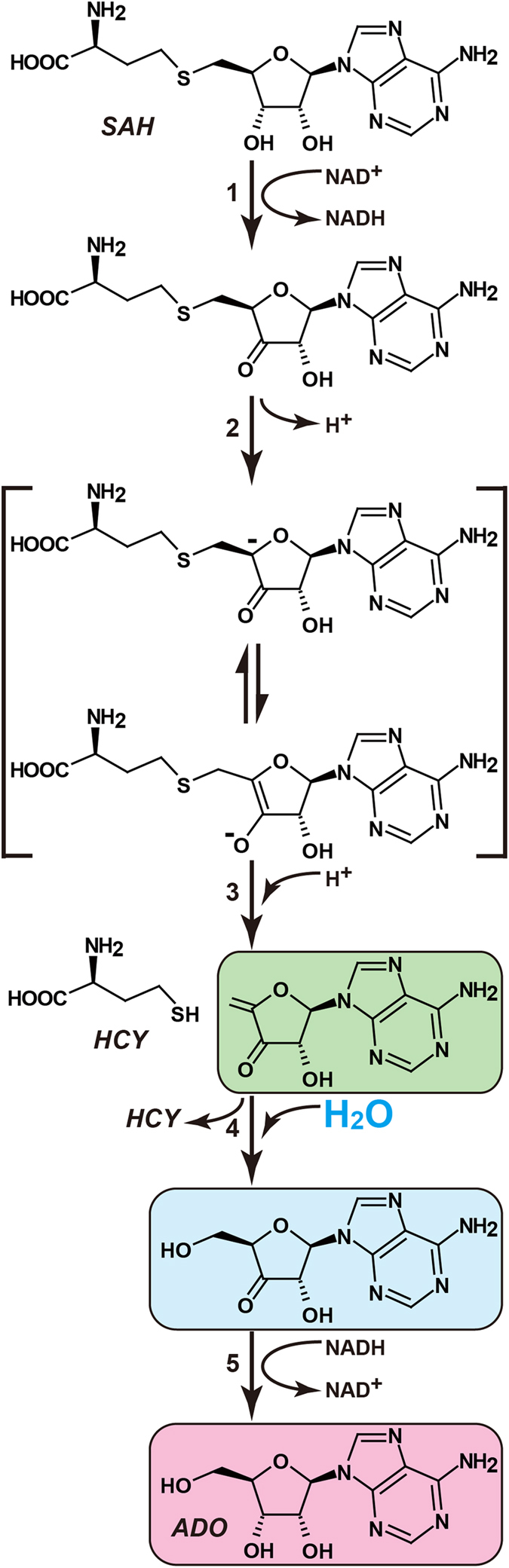
Possible reaction mechanism of the hydrolysis of SAH to HCY and ADO catalysed by SAHH. Our crystal structures provide structural information for the final three molecular stages of ADO formation by SAHH and are indicated by green, cyan, and pink.

**Figure 8:**
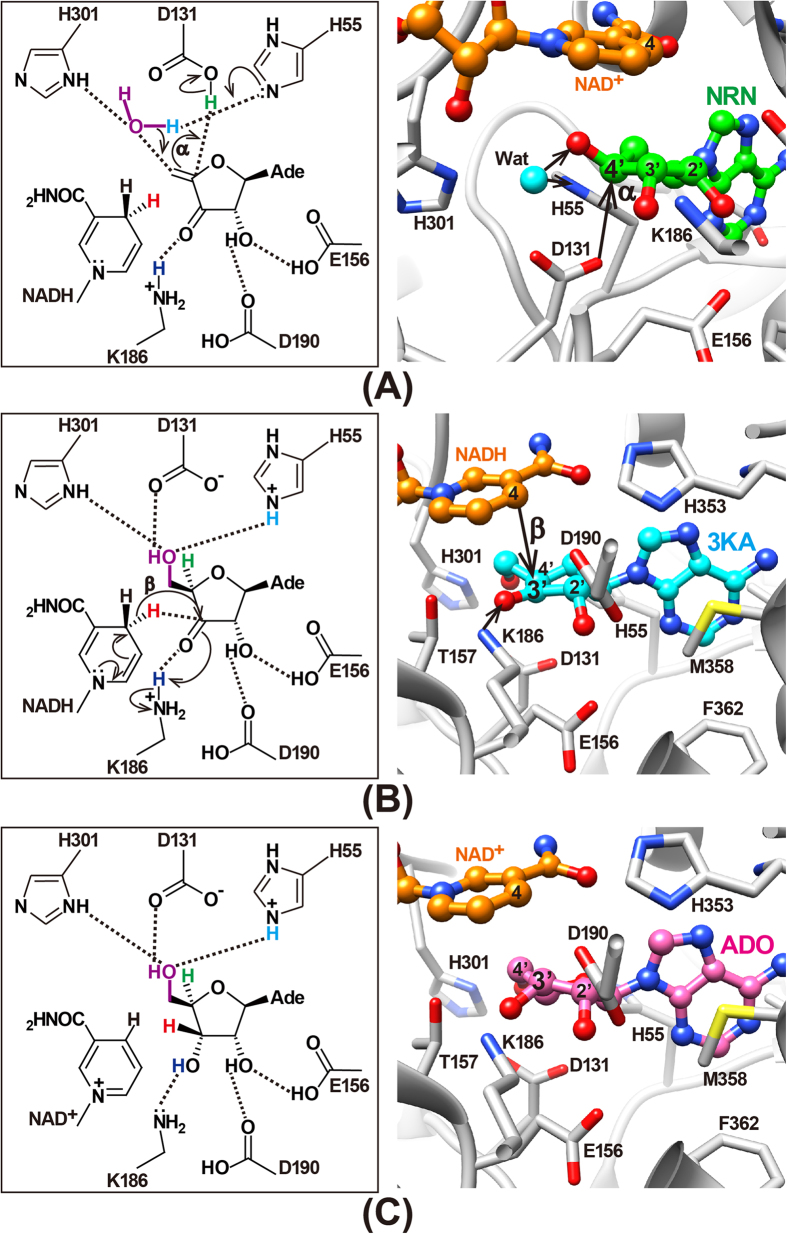
Structural validation of the last three steps of the proposed reaction mechanism for the hydrolysis of SAH to HCY and ADO catalysed by SAHH. Hydrogen atoms that transfer as proton ions are shown in blue, cyan, and green. Hydrogen that transfers as a hydride ion is shown in red. (**A**) Step 4: the addition of water to the C4′-C5′ double bond of 3′-keto-4′,5′-didehydroadenosine through a Michael-type addition (left). The MmSAHH/NAD^+^/NRN complex (right) is a structural homolog of this intermediate. (**B**) Step 5: the reduction of the 3′-keto group of 3′-keto ADO to form ADO (left). The MmSAHH/NADH/3KA complex (right) is a structural homolog of this intermediate. (**C**) Reaction product: the binding mode of the reaction product just after step 5 (left). The MmSAHH/NAD^+^/ADO complex (right) represents this state.

The mechanism of the reversible hydrolysis of SAH by SAHH was first fully described in 1979^33^, and kinetic studies have been reported in detail^34^,^35^,^36^. The reaction involves the oxidation-reduction of the tightly but not covalently bound cofactor NAD^+^. In the first step (Fig. 7, step 1), the 3′-OH group of the adenosine moiety of SAH is oxidised to a ketone by NAD^+^ and a general-base residue in the active site. The 3′-CH hydride and the 3′-OH proton of SAH are directly transferred to NAD^+^ and the base residue, respectively. In the second and third steps, the C4′ proton of the adenosine ribose is removed to form the carbanion intermediate (Fig. 7, step 2), followed by the release of HCY (Fig. 7, step 3). The fourth and fifth steps are the addition of water to the C4′-C5′ double bond through a Michael-type addition (Fig. 7, step 4) and the reduction of the 3′-keto group to form ADO (Fig. 7, step 5). The release of the ADO molecule induces a structural transition from the closed to the open conformation^23^. Our crystal structures of MmSAHH support the reaction mechanism proposed in 1979^33^ and are consistent with the more detailed reaction mechanism reported by Takusagawa and co-workers^22^. Additionally, this study provides novel crystallographic evidence that identifies some features of the reaction intermediates and the product (Figs 7 and 8).

In the NRN complex, a water molecule is observed adjacent to the 4′-OH group of NRN (Figs 3C and 5C). This water molecule occupies a position equivalent to that occupied by the 5′-OH group in the ADO complex and thus can be regarded as the donor in a Michael addition to the reaction intermediate 3′-keto-4′,5′-didehydroadenosine, as proposed in 1979^33^. It is likely that the NRN complex corresponds to step 4 of the hydrolytic reaction by SAHH. We focused on the roles of active site residues in the Michael addition (Fig. 8A, left). As proposed by Takusagawa and co-workers^22^, His55 acts as a general base to abstract a proton (cyan, left panel of Fig. 8A) from the bound water molecule and Asp131 donates the proton (green, left panel of Fig. 8A) to the C4′-carbon. His301 is involved in the stabilisation of the reaction intermediate. The roles of these residues are geometrically consistent with the crystal structure of the NRN complex (Fig. 8A, right). The side chains of His55 and His301 and the water molecule are located nearly parallel to the ribose ring. The side chain of Asp131 is located below the ribose ring and is suitable for donating a proton to the α side of the C4′-carbon. Therefore, we are confident that we have obtained the first direct evidence that a water molecule is hydrogen bonded to the side chains of His55 and His301 and is in a suitable position to act as the donor in a Michael addition to the reaction intermediate 3′-keto-4′,5′-didehydroadenosine as proposed in 1979^33^, even though the geometry of the ribosyl ring of NRN is somewhat different, i.e. 3′- and 4′-ribosyl carbons are not planar (Fig. 8A, right), than that of the true reaction intermediate. Examining the coordinate files of SAHHs in the Protein Data Bank (www.wwpdb.org), an equivalent water molecule is also found in the high-resolution structure of LlSAHH/NAD^+^/adenine complex^13^. In the adenine complex, the water molecule occupies a position equivalent to that occupied by the 5′-OH group in the LlSAHH/NAD^+^/ADO complex^13^ and forms hydrogen bonds with the side chains of His62, Asp139, and His350. The above mentioned water molecules observed in MmSAHH/NAD^+^/NRN and LlSAHH/NAD^+^/adenine complexes appear to be important for stabilising the flipped-over conformation by bridging His55 in the catalytic domain and His301 in the cofactor binding domain (MmSAHH numbering). These observations would contribute to the design of SAHH inhibitors, i.e., the position should be occupied by a polar group of the inhibitor.

The 3KA complex represents the binding mode of the reaction intermediate 3′-keto ADO after step 4, even though the ribosyl ring oxygen of ADO is replaced with a carbon atom in ARI (Fig. 2A,B). As previously proposed^22^, the final step of the hydrolysis reaction catalysed by SAHH proceeds as follows (Fig. 8B, left): a hydride ion (red, left panel of Fig. 8B) of NADH is directly transferred to the 3′-carbon, and the positively charged Lys186 acts as an acid and donates a proton (blue, left panel of Fig. 8B) to the 3′-keto oxygen of the bound 3′-keto ADO. The crystal structure of the 3KA complex is geometrically consistent with this mechanism (Fig. 8B, right). Position 4 of the nicotinamide ring of NADH is located over the ribose ring and is suitable for donating a hydride ion to the β side of the C3′′-carbon. The side chain of Lys186 is located below the ribose ring and is suitable for the formation of the 3′-OH group on the α side of the ribose ring.

In this crystal structure analysis, the co-crystallisation of MmSAHH with ARI resulted in oxidation of the 3′-OH group of the nucleoside to form 3KA (Fig. 3E). Thus, the synthetic reaction of SAHH appears to stall after the first step (the reverse direction of step 5 in Fig. 7). A 3KA complex has also been reported for MtSAHH^12^. As reported by Yang *et al*.^21^, a probable trigger for stabilising the 3′-keto form of the bound nucleoside appears to be a conformationally induced increase in the distance across which hydride transfer must occur between the C4 atom of the nicotinamide ring and the C3′ atom of the nucleoside. The distance was 3.2 Å in the case of 3′-hydroxy form (RnSAHH/NAD^+^/docked substrate), whereas 3.6 Å in the case of 3′-keto form (HsSAHH/NADH/3′-keto neplanocin A)^21^. Likewise, the corresponding distance increases from 3.2 Å (MmSAHH/NAD^+^/ADO) to 3.5 Å (MmSAHH/NADH/3KA). To better understand the stability of 3KA over ARI in the MmSAHH/NADH/3KA complex, we employed MM-GBSA scoring^37^ with the OPLS force field^38^ and the VSGB2.0 solvent model^39^. Table S3 shows that the MmSAHH complexed with 3KA (−96.533 kcal/mol) is more stable than that complexed with ARI (−82.516 kcal/mol), in agreement with the experimental observation that the crystallization of MmSAHH with ARI resulted in the 3KA complex. This explains the strong inhibitory activity of ARI as a mechanism-based inhibitor against SAHH enzymes from various species^40^,^41^. In contrast, in the present crystal structure analyses, 3′-keto forms were not observed for the ADO and NRN complexes. This may be explained by the reactivity of the 3′-keto carbon. In the case of ADO, the transfer of a hydride ion from position 4 of NADH to the 3′-keto carbon is facilitated by the presence of electronegative 2′- and 4′-oxygen atoms. The oxygen atoms are separated from the 3′-carbon by two bonds and make the 3′-carbon electropositive; thus, 3′-keto ADO can easily accept the hydride ion and convert to ADO. Similarly, the presence of 2′- and 4′-O (structurally equivalent to the 5′-carbon of ADO) atoms in NRN contribute to the 3′-keto to 3′-CHOH conversion. As described above, replacing the 4′-ribosyl oxygen with a less electronegative 5′-ribosyl carbon in ARI would lower the relative reactivity of the 3′-keto carbon and prevent the hydride transfer from NADH to the 3′-keto carbon. Thus, our observation of the 3′-keto form in the crystals of the ARI (Fig. 3E) complex but not the ADO (Fig. 3F) and NRN complexes is reasonable.

The ADO complex represents the binding mode of the reaction product immediately after step 5 (Fig. 8C). After the formation of ADO, the cleft between the catalytic domain and the cofactor-binding domain opens, and the ADO is released from the active site^23^,^24^,^26^.

**Figure 9:**
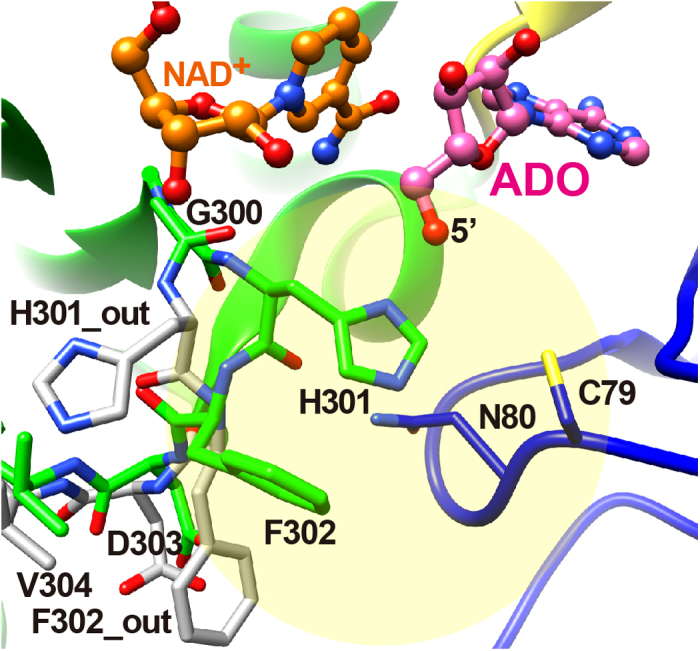
Dual conformations of His301 are observed in MmSAHH/NAD^+^/ADO complex . Flipped-out and flipped-over conformations of residues 300–304 are shown in grey and green, respectively. A putative access channel (HCY-binding site) is indicated by an ellipsoid. The Cys79 and Asn80 residues lining the putative access channel^12^ are marked.

In these crystal structure analyses, His301 forms a dual conformer, flipping out and flipping over, in the ADO complex (Fig. 5A). The difference in the flipped-out and flipped-over conformations is the result of a flip of the peptide plane between the His301 and Phe302 (Fig. 9); the presence of Gly300 facilitates this conformational change. The flexibility of this feature is correlated with the reaction mechanism of SAHH. The Gly300-His301-Phe302 sequence in MmSAHH is completely conserved among SAHHs from various species (Fig. 1). As reported in crystallographic studies of MtSAHH^12^, His363 corresponds to His301 in MmSAHH and regulates an access channel where the HCY moiety of the SAH substrate can bind (see Fig. 9). In the flipped-out conformation observed in the MtSAHH/NAD^+^/ethylthioadenosine complex, the active site can accommodate the HCY moiety of the substrate SAH, whereas in the flipped-over conformation observed in the MtSAHH/NAD^+^/ADO complex, there is no room for HCY to bind near the 5′-OH group of ADO. This is also true for the MmSAHH/NAD^+^/ADO complex. The putative access channel (HCY-binding site) is occupied by His301 and Phe302 in the flipped-over conformation (Fig. 9).

The conformational variability of the His residue (His301 in MmSAHH) has also been reported for SAHHs from various species. In the case of plant LlSAHH^13^, the flipped-out conformation of His350 corresponds to His301 in MmSAHH and His363 in MtSAHH and was observed in the LlSAHH/NAD^+^/ADO complex, whereas the flipped-over conformation was observed for the LlSAHH/NAD^+^/adenine complex. Similarly, the flipped-out conformation of the His residue was also observed for His345 in the PfSAHH/NAD^+^/ADO complex^11^. The conformational variability of the His residue can be summarised as follows. In the case of a tightly bound inhibitor complex, as in the ARI complex, the His residue adopts the flipped-over conformation. However, both the flipped-out and flipped-over conformations are observed for ADO complexes with the various SAHHs described above. This is because the bound ADO is not exposed to the solvent immediately after step 5 of the hydrolysis reaction (Fig. 7) but will then be exposed to the solvent for release as a reaction product. Our crystal structure shows the dual conformer of His301 in the ADO complex (Fig. 9), indicating a mixture of these two states. Furthermore, as expected, only the flipped-over conformations of His301 were observed in the 3KA and NRN complexes (Fig. 5B,C) because they are reaction intermediate analogues for ADO formation and they should be isolated from the solvent channel. Another explanation for the dual conformer of His301 in the ADO complex is that a possible hydrogen bond between flipped His55 and the ribosyl 4′-oxygen, not formed in the ARI and NRN complexes (the 4′-oxygen of ADO is replaced by a 5′-carbon atom), might alter the hydrogen-bond network consisting of His301, 5′-OH, and His55.

In this study, we report the high-resolution crystal structures of MmSAHH co-crystallised with ADO, ARI, and NRN at resolutions of 1.55, 1.55, and 1.65 Å, respectively. Co-crystallisation with ARI resulted in the oxidation of the 3′-OH group of the nucleoside to generate the 3′-keto form (3KA). The NRN complex is the first crystal structure of SAHH to be solved with NRN, and a water molecule is identified as the candidate donor in a Michael addition to the reaction intermediate 3′-keto-4′,5′-didehydroadenosine. These structures constitute the structural snapshots of the last three molecular stages in ADO formation and provide insights into the catalytic mechanism of SAHH.

## Methods

### Crystallisation and data collection

The purification and crystallisation of MmSAHH were carried out as described previously^42^. The purification of MmSAHH was performed by affinity chromatography using a TALON column and gel-filtration chromatography using a Superdex 200 pg column. The absorption spectrum of the purified and concentrated MmSAHH showed no prominent shoulder around 340 nm, indicating that the sample contained little NADH-bound form of the enzyme. The crystals of the MmSAHH/NAD^+^/ADO (or analogue) complexes were prepared as follows. ADO, ARI, and RBV were purchased from Sigma-Aldrich (St Louis, Missouri, USA). NRN was synthesised as described^43^. ADO or its analogues (ARI, NRN, or RBV, see Fig. 2) were dissolved in a standard buffer (0.1-M sodium chloride in 0.05-M Tris-HCl buffer at pH 7.4) to a concentration of 20 mM. The 15-mg/ml MmSAHH solution was mixed with aliquots of the respective ligand solutions in a volume ratio of 4:1 with a final ligand concentration of 4 mM and a MmSAHH concentration of 12 mg/ml. A droplet was prepared by mixing an equal volume of the working solution described above and the reservoir solution containing 0.2-M sodium formate and 22%(w/v) PEG3350 in Hepes/NaOH buffer at pH 7.0. The droplet was suspended over 500 μl of reservoir solution in a 24-well plate. The crystals belong to an orthorhombic space group *I*222 with cell dimensions of *a*  = 100.64 Å, *b* = 104.44 Å, and *c* = 177.31 Å for the ADO complex. Crystals of the ADO analogue complexes were isomorphous with the crystals of the ADO complex. Assuming two subunits (a half tetramer) per asymmetric unit, we obtained a V_M_ value of 2.43 Å^3^/Da, corresponding to a solvent content of 49.6%. For data collection under cryogenic conditions, the crystals in a droplet were directly transferred to a harvesting solution [0.2-M sodium formate, 22%(w/v) PEG3350, and 20%(v/v) glycerol in Hepes/NaOH buffer at pH 7.0] for 1 minute. Crystals were mounted in nylon loops and flash-frozen in a cold nitrogen-gas stream at 100 K immediately prior to data collection. Data collection was performed at 100 K using a CCD detector with the synchrotron radiation of the Advanced Ring of the Photon Factory (PF-AR). The data were processed using the HKL-2000 package^44^. The data collection statistics are summarised in Table S1.

### Structure determination and refinement

The initial phase determination was performed with the molecular replacement method using one protomer of HsSAHH^9^ (PDB code: 1LI4) as a search model. Cross-rotation and translation functions were calculated using the program MOLREP^45^ from the CCP4 suite^46^ for the ADO complex. Automatic model building and refinement were carried out using the programs ARP/wARP^47^ and REFMAC5^48^, and further iterative manual model building and refinement were performed with the programs XtalView^49^ and REFMAC5. The refined structure of the MmSAHH/NAD^+^/ADO complex was then used for the structure determination of the other ADO analogue complexes by the difference Fourier method. The occupancies of the dual conformations for residues 300–304 observed in the ADO and RBV complexes were manually refined by varying the ratio of the flipped-out and flipped-over conformations. The refinement statistics are summarised in Table S2.

### Physics-based scoring

To better understand the stability of 3KA over ARI in the MmSAHH/NADH/3KA complex, we employed MM-GBSA scoring^37^ with the OPLS force field^38^ and the VSGB2.0 solvent model^39^. All computations were carried out through Maestro version 10.1 (Schrödinger, LLC, New York, USA). To computationally model the complex with ARI, we simply substituted the carbonyl oxygen of 3KA in the complex with a hydroxyl oxygen. Before the MM-GBSA scoring, both the experimentally determined MmSAHH/NADH/3KA and the MmSAHH/NAD^+^/ARI model complexes were energy minimized with the default parameters in the protein preparation wizard^50^.

### Graphical programs

Figures 1,2 and 7 were produced with the program Adobe Illustrator (Adobe Systems Inc., San Jose, California, USA). Figure 3 was produced with the programs XtalView and Raster3D^51^. Figures 4, 5, 6,8 and 9 were produced with the programs UCSF Chimera^52^ and Adobe Illustrator. Supplementary information. Supplementary Tables S1-S3.

## Additional Information

**Accession codes:** Atomic coordinates for the reported structures have been deposited in the Protein Data Bank under accession codes 5AXA (ADO complex), 5AXB (NRN complex), 5AXC (3KA complex), and 5AXD (RBV complex).

**How to cite this article**: Kusakabe, Y. *et al*. Structural insights into the reaction mechanism of *S*-adenosyl-L-homocysteine hydrolase. *Sci. Rep.* **5**, 16641; doi: 10.1038/srep16641 (2015).

## Supplementary Material

Supplementary Information

## References

[b1] Cantoni G. L. Biological methylation: selected aspects. Annu. Rev. Biochem. 44, 435–451 (1975). 1094914 10.1146/annurev.bi.44.070175.002251

[b2] Tehliverts O., Malanovic N., Visram M., Pavkov-Keller T. & Keller W. *S*-adenosyl-L-homocysteine hydrolase and methylation disorders: Yeast as a model system. Biochim. Biophys. Acta 1832, 204–215 (2013). 23017368 10.1016/j.bbadis.2012.09.007 PMC3787734

[b3] Dayal S. . Endothelial dysfunction and elevation of *S*-adenosylhomocysteine in cystathionine β-synthase deficient mice. Circ. Res. 88, 1203–1209 (2001). 11397788 10.1161/hh1101.092180

[b4] Baric I. Inherited disorders in the conversion of methionine to homocysteine. J. Inherit. Metab. Dis. 32, 459–471 (2009). 19585268 10.1007/s10545-009-1146-4

[b5] Teng Y. W., Mehedint M. G., Garrow T. A. & Zeisel S. H. Deletion of betaine-homocysteine *S*-methyltransferase in mice perturbs choline and 1-carbon metabolism, resulting in fatty liver and hepatocellular carcinomas. J. Biol. Chem. 286, 36258–36267 (2011). 21878621 10.1074/jbc.M111.265348 PMC3196139

[b6] Leal J. F. . *S*-adenosylhomocysteine hydrolase downregulation contributes to tumorigenesis. Carcinogenesis 29, 2089–2095 (2008). 18713839 10.1093/carcin/bgn198

[b7] Herrmann W. . Disturbed homocysteine and methionine cycle intermediates *S*-adenosylhomocysteine and *S*-adenosylmethionine are related to degree of renal insufficiency in type 2 diabetes. Clin. Chem. 51, 891–897 (2005). 15774574 10.1373/clinchem.2004.044453

[b8] Poirier L. A. . Blood *S*-adenosylmethionine concentrations and lymphocyte methylenetetrahydrofolate reductase activity in diabetes mellitus and diabetic nephropathy. Metabolism 50, 1014–1018 (2001). 11555831 10.1053/meta.2001.25655

[b9] Turner M. A. . Structure determination of selenomethionyl *S*-adenosylhomocysteine hydrolase using data at a single wavelength. Nat. Struct. Biol . 5, 369–376 (1998). 9586999 10.1038/nsb0598-369

[b10] Hu Y. . Crystal structure of *S*-adenosylhomocysteine hydrolase from rat liver. Biochemistry 38, 8323–8333 (1999). 10387078 10.1021/bi990332k

[b11] Tanaka N. . Crystal structure of *S*-adenosyl-L-homocysteine hydrolase from the human malaria parasite *Plasmodium falciparum*. J. Mol. Biol. 343, 1007–1017 (2004). 15476817 10.1016/j.jmb.2004.08.104

[b12] Reddy M. C. . Crystal structures of *Mycobacterium tuberculosis S*-adenosyl-L-homocysteine hydrolase in ternary complex with substrate and inhibitors. Protein Sci. 17, 2134–2144 (2008). 18815415 10.1110/ps.038125.108 PMC2590921

[b13] Brzezinski K., Dauter Z. & Jaskolski M. High-resolution structures of complexes of plant *S*-adenosyl-L-homocysteine hydrolase (*Lupinus luteus*). Acta Crystallogr . D68, 218–231 (2012). 10.1107/S0907444911055090 PMC3282620 22349223

[b14] Matuszewska B. & Borchardt R. T. The mechanism of inhibition of *Alcaligenes faecalis S*-adenosylhomocysteine hydrolase by neplanocin A. Arch. Biochem. Biophys. 256, 50–55 (1987). 3606132 10.1016/0003-9861(87)90424-3

[b15] Chiang P. K. Biological effects of inhibitors of *S*-adenosylhomocysteine hydrolase. Pharmacol. Ther. 77, 115–134 (1988). 9578320 10.1016/s0163-7258(97)00089-2

[b16] Zhang Y. M. . Synthesis and biological evaluation of immunosuppressive agent DZ2002 and its stereoisomers. Bioorg. Med. Chem. 16, 9212–9216 (2008). 18815049 10.1016/j.bmc.2008.09.017

[b17] Langheinrich A. C. . Effects of 3-deazaadenosine on homocysteine and atherosclerosis in apolipoprotein E-deficient mice. Atherosclerosis 171, 181–192 (2003). 14644386 10.1016/j.atherosclerosis.2003.08.028

[b18] Komoto J. . Effects of site-directed mutagenesis on structure and function of recombinant rat liver *S*-adenosylhomocysteine hydrolase. Crystal structure of D244E mutant enzyme. J. Biol. Chem. 275, 32147–32156 (2000). 10913437 10.1074/jbc.M003725200

[b19] Huang Y. . Inhibition of *S*-adenosylhomocysteine hydrolase by acyclic sugar adenosine analogue D-eritadenine. Crystal structure of *S*-adenosylhomocysteine hydrolase complexed with D-eritadenine. J. Biol. Chem. 277, 7477–7482 (2002). 11741948 10.1074/jbc.M109187200

[b20] Takata Y. . Catalytic mechanism of *S*-adenosylhomocysteine hydrolase. Site-directed mutagenesis of Asp-130, Lys-185, Asp-189, and Asn-190. J. Biol. Chem. 277, 22670–22676 (2002). 11927587 10.1074/jbc.M201116200

[b21] Yang X. . Catalytic strategy of *S*-adenosyl-L-homocysteine hydrolase: transition-state stabilization and the avoidance of abortive reactions. Biochemistry 42, 1900–1909 (2003). 12590576 10.1021/bi0262350

[b22] Yamada T. . Catalytic mechanism of *S*-adenosylhomocysteine hydrolase: roles of His 54, Asp130, Glu155, Lys185, and Aspl89. Int. J. Biochem. Cell Biol. 37, 2417–2435 (2005). 16061414 10.1016/j.biocel.2005.06.009

[b23] Wang M., Borchardt R. T., Schowen R. L. & Kuczera K. Domain motions and the open-to-closed conformational transition of an enzyme: a normal mode analysis of *S*-adenosyl-L-homocysteine hydrolase. Biochemistry 44, 7228–7239 (2005). 15882061 10.1021/bi047524m

[b24] Wang M. . Effects of ligand binding and oxidation on hinge-binding motions in *S*-adenosyl-L-homocysteine hydrolase. Biochemistry 45, 7778–7786 (2006). 16784229 10.1021/bi0523106

[b25] Yamada T. . Structure and function of eritadenine and its 3-deaza analogues: potent inhibitors of *S*-adenosylhomocysteine hydrolase and hypocholesterolemic agents. Biochem. Pharmacol. 73, 981–989 (2007). 17214973 10.1016/j.bcp.2006.12.014

[b26] Hu C., Fang J., Borchardt R. T., Schowen R. L. & Kuczera K. Molecular dynamics simulations of domain motions of substrate-free *S*-adenosyl-L-homocysteine hydrolase in solution. Proteins 71, 131–143 (2008). 17932938 10.1002/prot.21664

[b27] Fabianowska-Majewska K., Duley J. A. & Simmonds H. A. Effects of novel anti-viral adenosine analogues on the activity of *S*-adenosylhomocysteine hydrolase from human liver. Biochem. Pharmacol. 48, 897–903 (1994). 8093102 10.1016/0006-2952(94)90360-3

[b28] Cai S., Li Q.-S., Borchardt R. T., Kuczera K. & Schowen R. L. The antiviral drug ribavirin is a selective inhibitor of *S*-adenosyl-L-homocysteine hydrolase from *Trypanosoma cruzi*. Bioorg. Med. Chem. 15, 7281–7287 (2007). 17845853 10.1016/j.bmc.2007.08.029 PMC3830956

[b29] Bougie I. & Bisaillon M. The broad spectrum antiviral nucleoside ribavirin as a substrate for a viral RNA capping enzyme. J. Biol. Chem. 279, 22124–22130 (2004). 15037606 10.1074/jbc.M400908200

[b30] Zheng Y. . Crystal structures of *S*-adenosylhomocysteine hydrolase from the thermophilic bacterium *Thermotoga maritima*. J. Struct. Biol. 190, 135–142 (2015). 25791616 10.1016/j.jsb.2015.03.002

[b31] Harding M. M. Metal-ligand geometry relevant to proteins and in proteins: sodium and potassium. Acta Crystallogr . D58, 872–874 (2002). 10.1107/s0907444902003712 11976508

[b32] Harding M. M. Small revisions to predicted distances around metal sites in proteins. Acta Crystallogr . D62, 678–682 (2006). 10.1107/S0907444906014594 16699196

[b33] Palmer J. L. & Abeles R. H. The mechanism of action of *S*-adenosylhomocysteinase. J. Biol. Chem. 254, 1217–1226 (1979). 762125

[b34] Porter D. J. & Boyd F. L. Mechanism of bovine liver *S*-adenosylhomocysteine hydrolase. Steady-state and pre-steady-state kinetic analysis. J. Biol. Chem. 266, 21616–21625 (1991). 1939191

[b35] Porter D. J. & Boyd F. L. Reduced *S*-adenosylhomocysteine hydrolase. Kinetics and thermodynamics for binding of 3′-ketoadenosine, adenosine, and adenine. J. Biol. Chem. 267, 3205–3213 (1992). 1737776

[b36] Porter D. J. *S*-adenosylhomocysteine hydrolase. Stereochemistry and kinetics of hydrogen transfer. J. Biol. Chem. 268, 66–73 (1993). 8416969

[b37] Lyne P. D., Lamb M. L. & Saeh J. C. Accurate prediction of the relative potencies of members of a series of kinase inhibitors using molecular docking and MM-GBSA scoring. J. Med. Chem. 49, 4805–4808 (2006). 16884290 10.1021/jm060522a

[b38] Jorgensen W. L., Maxwell D. S. & Tirado-Rives J. Development and testing of the OPLS all-atom force field on conformational energetics and properties of organic liquids. J. Am. Chem. Soc. 118, 11225–11236 (1996).

[b39] Li J. . The VSGB 2.0 model: a next generation energy model for high resolution protein structure modeling. Proteins 79, 2794–2812 (2011). 21905107 10.1002/prot.23106 PMC3206729

[b40] Guranowski A., Montgomery J. A., Cantoni G. L. & Chiang P. K. Adenosine analogues as substrates and inhibitors of *S*-adenosylhomocysteine hydrolase. Biochemistry 20, 110–115 (1981). 7470463 10.1021/bi00504a019

[b41] De Clercq E. Carbocyclic adenosine analogues as *S*-adenosylhomocysteine hydrolase inhibitors and antiviral agents: recent advances. Nucleosides Nucleotides 17, 625–634 (1998). 9708366 10.1080/07328319808005205

[b42] Ishihara M. . Crystallization of mouse *S*-adenosyl-L-homocysteine hydrolase. Acta Crystallogr . F66, 313–315 (2010). 10.1107/S1744309110000771 PMC2833045 20208169

[b43] Kitade Y., Kozaki A., Miwa T. & Nakanishi M. Synthesis of base-modified noraristeromycin derivatives and their inhibitory activity against human and *Plasmodium falciparum* recombinant *S*-adenosyl-L-homocysteine hydrolase. Tetrahedron 58, 1271–1277 (2002).

[b44] Otwinowski Z. & Minor W. Processing of X-ray diffraction data collected in oscillation mode. Methods in Enzymol . 276, 307–326 (1997). 10.1016/S0076-6879(97)76066-X 27754618

[b45] Vagin A. & Teplyakov A. Molecular replacement with MOLREP. Acta Crystallogr . D66, 22–25 (2010). 10.1107/S0907444909042589 20057045

[b46] Collaborative Computational Project Number 4. The CCP4 suite: programs for protein crystallography. Acta Crystallogr . D50, 760–763 (1994). 10.1107/S0907444994003112 15299374

[b47] Langer G., Cohen S. X., Lamzin V. S. & Perrakis A. Automated macromolecular model building for X-ray crystallography using ARP/wARP version 7. Nat. Protoc. 3, 1171–1179 (2008). 18600222 10.1038/nprot.2008.91 PMC2582149

[b48] Murshudov G. N. . REFMAC5 for the refinement of macromolecular crystal structures. Acta Crystallogr . D67, 355–367 (2011). 10.1107/S0907444911001314 PMC3069751 21460454

[b49] McRee D. E. XtalView/Xfit – A versatile program for manipulating atomic coordinates and electron density. J. Struct. Biol. 125, 156–165 (1999). 10222271 10.1006/jsbi.1999.4094

[b50] Sastry G. M., Adzhigirey M., Day T., Annabhimoju R. & Sherman W. Protein and ligand preparation: parameters, protocols, and influence on virtual screening enrichments. J. Comput. Aided Mol. Des. 27, 221–234 (2013). 23579614 10.1007/s10822-013-9644-8

[b51] Merritt E. A. & Murphy M. E. P. Raster3D Version 2.0: A program for photorealistic molecular graphics. Acta Crystallogr . D50, 869–873 (1994). 10.1107/S0907444994006396 15299354

[b52] Pettersen E. F. . UCSF Chimera – a visualization system for exploratory research and analysis. J. Comput. Chem. 25, 1605–1612 (2004). 15264254 10.1002/jcc.20084

