# Supplementary material for: Structural insights into the reaction mechanism of S-adenosyl-L-homocysteine hydrolase
Source: Sci Rep. 2015 Nov 17;5:16641. doi: 10.1038/srep16641 (PMC4647836; doi:10.1038/srep16641)
Supplement: Supplementary Information [file srep16641-s1.pdf]

SUBJECT AREAS: ENZYME MECHANISMS, STRUCTURAL BIOLOGY

Correspondence and requests for materials should be addressed to
N.T. (ntanaka@pharm.showa-u.ac.jp)

Structural insights into the reaction mechanism of *S*-adenosyl-L-homocysteine hydrolase

Yoshio Kusakabe[1,4], Masaaki Ishihara[1,4], Tomonobu Umeda[1], Daisuke Kuroda[1], Masayuki Nakanishi[2], Yukio Kitade[3], Hiroaki Gouda[1], Kazuo T. Nakamura[1], and Nobutada Tanaka[1]

[1]School of Pharmacy, Showa University, 1-5-8 Hatanodai, Shinagawa-ku, Tokyo 142-8555, Japan; [2]College of Pharmaceutical Sciences, Matsuyama University, 4-2 Bunkyo-cho, Matsuyama, Ehime 790-8578, Japan; [3]Faculty of Engineering, Gifu University, 1-1 Yanagido, Gifu, Gifu 501-1193, Japan

[4]These authors contributed equally to this work.

Supplementary information includes:

Supplementary Tables S1-S3

Table S1 | Data collection statistics for the MmSAHH/nucleoside complexes.

Data set	ADO	NRN	3KA	RBV
Facility<br>Beamline	PF-AR*<br>NW12A	PF-AR<br>NE3A	PF-AR<br>NE3A	PF-AR<br>NE3A
Detector	ADSC Q210r	ADSC Q270	ADSC Q270	ADSC Q270
Wavelength (Å)	1.0000	1.0000	1.0000	1.0000
Space group	*I*222	*I*222	*I*222	*I*222
No. of subunits /ASU	2<br>(1/2 tetramer)	2<br>(1/2 tetramer)	2<br>(1/2 tetramer)	2<br>(1/2 tetramer)
Solvent content (%)	49.6	49.4	46.8	49.6
Cell				
a (Å)	100.644	100.442	98.202	100.580
b (Å)	104.443	104.459	102.881	104.612
c (Å)	177.309	176.744	174.827	177.007
Resolution (Å)<br>(outer shell)	1.55<br>(1.58-1.55)	1.65<br>(1.68-1.65)	1.55<br>(1.58-1.55)	1.60<br>(1.63-1.60)
No. of observed reflections	544,781	707,961	732,274	661,725
No. of unique reflections	132,732	109,318	125,615	121,803
Multiplicity	4.1 (3.7)	6.5 (2.6)	5.8 (4.0)	5.4 (4.1)
$I / \sigma(I)$	30.0 (2.9)	40.2 (2.1)	44.7 (2.5)	28.5 (3.5)
B-factor (Å^2)	19.6	21.9	21.8	13.9
R_{sym} (%)	9.9 (57.4)	8.2 (47.8)	8.8 (51.3)	9.9 (47.4)
Completeness (%)	98.7 (99.8)	98.5 (82.4)	98.4 (90.8)	99.6 (99.6)

*Photon Factory Advanced Ring

Table S2 | Refinement statistics for the MmSAHH/nucleoside complexes.

Data set	ADO	NRN	3KA	RBV
PDB ID	5AXA	5AXB	5AXC	5AXD
Resolution range (Å) (outer shell)	50 - 1.55 (1.59 - 1.55)	50 - 1.65 (1.69 - 1.65)	50 - 1.55 (1.59 - 1.55)	50 - 1.60 (1.64 - 1.60)
No. of reflections				
working set	123,140	103,797	118,147	115,300
test set	6,521	5,459	6,227	6,107
R-factor	0.188	0.154	0.179	0.159
Free *R*-factor	0.229	0.181	0.210	0.180
No. of protein atoms* (avg. *B*-factors (Å^2))	6,730 (18.4)	6,650 (19.9)	6,650 (20.8)	6,730 (12.4)
No. of NAD(H) atoms (avg. *B*-factors (Å^2))	88 (2 x 44) (15.4)	88 (2 x 44) 15.4	88 (2 x 44) 15.6	88 (2 x 44) 7.4
No. of nucleoside atoms (avg. *B*-factors (Å^2))	38 (2 x 19) (16.0)	36 (2 x 18) (16.7)	38 (2 x 19) (15.8)	34 (2 x 17) (10.8)
No. of Na ions (avg. *B*-factors (Å^2))	2 (2 x 1) (21.6)	2 (2 x 1) (25.7)	2 (2 x 1) (26.1)	-
No. of water molecules (avg. *B*-factors (Å^2))	1,134 (33.0)	956 (34.9)	943 (33.8)	1,073 (27.6)
RMSD				
bond distances (Å)	0.015	0.014	0.016	0.012
bond angles (°)	1.655	1.494	1.722	1.448
Dual conformations for residues 300-304 (flipping out : flipping over)	0.3 : 0.7	0.0 : 1.0	0.0 : 1.0	0.6 : 0.4

*There are two unique subunits in the ASU. The N-terminal residues (residues 1-3 and 1-2 in subunits A and B, respectively) are disordered. Residues 300-304 in both subunits of ADO and RBV complexes exhibit dual conformations (flipping out vs. flipping over). The flipped-out : flipped-over ratios are shown in the bottom row.

Table S3 | Physics-based scoring of experimentally determined complex (MmSAHH/NADH/3KA) and model complex (MmSAHH/NAD^+/ARI).

	Prime energy (kcal / mol)	MM-GBSA dG bind (kcal / mol)
MmSAHH / NADH / 3KA (X-ray structure)	-18,960.1	-96.533
MmSAHH / NAD^+ / ARI (model structure)	-18,926.9	-82.516
